# Neonatal Resuscitation Practices in Romania: A Survey of the Romanian Association of Neonatology (ANR) and the Union of European Neonatal and Perinatal Societies (UENPS)

**DOI:** 10.2478/jccm-2024-0010

**Published:** 2024-01-30

**Authors:** Manuela Cucerea, Marta Simon, Silvia Maria Stoicescu, Ligia Daniela Blaga, Radu Galiș, Maria Stamatin, Gabriela Olariu, Maria Livia Ognean

**Affiliations:** George Emil Palade University of Medicine, Pharmacy, Science, and Technology of Targu Mures, Romania; Carol Davila University of Medicine and Pharmacy, Bucuresti, Romania; Iuliu Hațieganu University of Medicine and Pharmacy, Cluj-Napoca, Romania; Oradea County Emergency Hospital, Oradea, Romania; Grigore T Popa University of Medicine and Pharmacy, Iasi, Romania; Emergency County Hospital, Timisoara, Romania; Faculty of Medicine, Lucian Blaga University of Sibiu, Sibiu, Romania

**Keywords:** Romania, delivery room, neonatal resuscitation, survey

## Abstract

**Introduction:**

This study is part of a European survey on delivery room practices endorsed by the Union of European Neonatal and Perinatal Societies (UENPS) and the Romanian Association of Neonatology (ANR). The aim of our study was to evaluate the current neonatal resuscitation practices in Romanian maternity hospitals and to compare the results between level III and level II centers.

**Material and Methods:**

The questionnaire was distributed through ANR by email link to heads of neonatal departments of 53 Romanian maternity hospitals with more than one thousand of births per year between October 2019 and September 2020, having 2018 as the reference year for data collection.

**Results:**

The overall response rate to the questionnaire was 62.26% (33/53), 83.33% (15/18) for level-III centers and 51.43% (18/35) for level-II centers. Of the responding centers, 18 (54,54%) were academic hospitals, 15 (83,33%) were level III and 3 (16,67%) level II hospitals. In 2018, responding centers reported 81.139 births representing 42.66% of all Romanian births (190.170). There were significant differences between level-III and level-II maternity hospitals regarding the number of births in 2018 (3028.73±1258.38 vs 1983.78±769.99; P=0.006), lowest GA of routinely assisted infants in delivery room (25.07±3.03 weeks vs 30.44±3.28, P<0.001), inborn infants with BW<1500 admitted to neonatal intensive care unit (NICU) in 2018 (66.86±39.14 g vs 22.87±31.50 g; P=0.002), and antenatal counseling of parents before the delivery of a very preterm infant or an infant with expected problems (60% vs 22.2%; P=0.027). There were no significant differences of thermal and umbilical cord management, positive pressure delivery, heart rate assessment between responding centers.

**Conclusion:**

The adherence to new guidelines was high among responding centers regarding thermal and umbilical cord management, initial FiO_2_, but aspects like antenatal counseling, EKG monitoring, laryngeal mask, and heated/humidified gases availability and administration, and simulation-based training require further implementation.

## Introduction

Political changes and socio-economic development in Romania since 1990 together with the implementation of public health measures were associated with important decreasing trends in neonatal mortality from 8.9 deaths per thousand live births in 1990 to 3.1 deaths per thousand live births in 2021 [1,2]. A significant reduction in neonatal mortality can be achieved by immediate assessment and prompt and correct resuscitation of the newborn at birth, especially the high-risk ones. The first minutes after birth (“golden minute”) is crucial for establishment of breathing and oxygenation, with major impact on survival and long-term outcome of the newborn [3]. Thus, the implementation of new evidence-based interventions in neonatal resuscitation, such including support of physiologic transition from intrauterine to extrauterine life, umbilical cord management at birth, achievement of neutral thermal environment, titration of the inspiratory fraction of oxygen, the use of non-invasive techniques of respiratory support should be o priority all over the world [4]. The Romanian Association of Neonatology developed and continuously reviewed national guidelines in accordance with the updated American Academy of Pediatrics (AAP)/American Heart Association (AHA) guidelines and International Liaison Committee on Resuscitation (ILCOR) recommendations.

According to the Romanian Standards of Perinatal Care (2002) there are three levels of maternity hospitals classified depending on the level of competence: level I (healthy term infants), level II (healthy and sick full-term and preterm infants over 32 weeks of gestational age who do not require invasive ventilation) and level III (regional centers) where critical infants requiring intensive care and treatment and invasive respiratory support are cared for, especially preterm infants under 32 weeks of gestational age.

The aim of our study was to evaluate the current neonatal resuscitation practices in Romanian maternity hospitals with more than one thousand of births per year and to compare the results between level III and level II centers.

## Methods

This study is part of a European survey on delivery room practices endorsed by the Union of European Neonatal and Perinatal Societies (UENPS) and the Romanian Association of Neonatology (ANR). The survey was approved by Padua Provincial Institutional Review Board (protocol # 0035420) and declared as not meeting the criteria for human subject research. The cross-sectional electronic survey was based on a 91-item structured questionnaire elaborated by a committee of experts on neonatal resuscitation and members of the task force of the Neonatal Resuscitation of the Italian Society of Neonatology, having 2018 as the reference year for data collection [5]. The questionnaire was anonymous and contained questions about epidemiological data, perinatal organizational aspects, medical equipment, initial steps interventions, ventilation and oxygen therapy, ethics, and education [5,6,7]. The questionnaire was distributed through ANR by email link (www.surveymonkey.com) to heads of neonatal departments of 53 Romanian maternity hospitals with more than one thousand of births per year between October 2019 and September 2020. The answer to the questionnaire was considered as informed consent of the participants. A reminder was sent to nonresponders for 3 times [5].

### Statistical analysis

Statistical analysis was performed using IBM SPSS 23. Means plus standard deviations (SD) were calculated for numerical variables and compared using Independent Samples t-test. Frequencies and proportions (n, %) were used to describe categorical data. Pearson chi-square or Fisher’s exact tests were used to compare variables between centers.

## Results

The overall response rate to the questionnaire was 62.26% (33/53), 83.33% (15/18) for level-III centers and 51.43% (18/35) for level-II centers. Of the responding centers, 18 (54,54%) were academic hospitals, 15 (83,33%) were level III and 3 (16,67%) level II hospitals. In 2018, responding centers reported 81.139 births representing 42.66% of all Romanian births (190.170) (8). The results of the survey are reported in Tables 1–6.

**Table 1.** Characteristics of centers and organizational aspects before birth

	**Total No (%)**	**Level III maternity hospital (n=15)**	**Level II maternity hospital (n=18)**	**p**
**Maternity hospital**	33	15	18	
**Academic hospital**	18	15	3	
Number of births 2018	81139	45431	35708	0.006
		3028.73±1258.38	1983.78±769.99	
Lowest GA of routinely assisted infants in Delivery Room (DR)	33 (100)	25.07±3.03 (23–32)	30.44±3.28 (23–33)	<0.001
		Median 24	Median 32	
Inborn infants with BW<1500 admitted to NICU in 2018/No of reporting centres	23 (69.7)	66.86±39.14	22.87±31.50	0.002
		14/15 (93.33)	15/18 (83.33)	
Outborn infants with BW<1500 admitted to NICU in 2018/No of reporting centres	20 (60.1)	15.50±11.02	8.93±14.99	0.193
		14/15 (93.33)	15/18 (83.33)	
Antenatal counseling before the delivery	13/33 (39.4)	9/15 (60.0)	4/18 (22.2)	0.027
**Available facilities in maternity hospitals**				
Nasal CPAP/HFNC	33 (100)	15/15 (100)	18/18 (100)	NA
Mechanical ventilation (MV)	29 (87.9)	15/15 (100)	14/18 (77.8)	0.229
High frequency oscillatory ventilation (HFOV)	20 (60.6)	12/15 (80.0)	8/18 (44.4)	0.041
Inhaled nitric oxide (iNO)	7 (21.2)	7/15 (46.7)	0/18 (0)	0.002
Therapeutic hypothermia	10 (30.3)	8/15 (53.3)	2/18 (11.1)	0.012
CPAP in delivery room	33 (100)	15/15 (100)	18/18 (100)	NA
ECMO	0 (0)	0/15 (0)	0/18 (0)	NA
Resuscitation team present at every delivery	27 (81.8)	12/15 (80)	15/18 (83.3)	0.459
**Routinely resuscitation team for a low-risk delivery**				
Pediatrician/neonatologist	29 (87.9)	13/15 (86.7)	16/18 (88.9)	0.626
Obstetrician	3 (9.1)	1/15 (6.7)	2/18 (11.1)	0.570
Anesthesiologist	1 (3.0)	0/15 (0)	1/18 (5.6)	0.545
Nurse	6 (18.2)	2/15 (13.3)	4/18 (22.2)	0.423
Midwife	32 (97)	15/15 (100)	17/18 (94.4)	0.545
**Routinely resuscitation team for a high-risk delivery**				
Pediatrician/neonatologist	33 (100)	15/15 (100)	18/18 (100)	NA
Obstetrician	4 (12.1)	2/15 (13.3)	2/18 (11.1)	0.626
Anesthesiologist	3 (9.1)	1/15 (6.7)	2/18 (11.1)	0.570
Nurse	6 (18.2)	3/15 (20.0)	3/18 (16.7)	0.577
Midwife	33 (100)	15/15 (100)	18/18 (100)	NA
**Resuscitation team members qualified with full resuscitation skills**				
Pediatrician/neonatologist	33 (100)	15/15 (100)	18/18 (100)	NA
Obstetrician	0 (0)	0/15 (0)	0/18 (0)	NA
Anesthesiologist	8 (24.2)	3/15 (20.0)	5/18 (27.38)	0.459
Nurse	0 (0)	0/15 (0)	0/18 (0)	NA
**Gestational age limit for initiating full resuscitation at birth**				
Yes	19 (57.6)	15/15 (100.0)	18/18 (100)	NA
Range	20 (60.6)	22–32	22–32	-
Median gestational age	20 (60.6)	24	24	NA
Gestational age limit /No of reporting centres	20 (60.6)	23.53±0.74	24.44±2.04	0.111
**Time limit to stop full resuscitation in severely asphyxiated infant**				
Yes	24 (72.7)	11/15 (73.3)	13/18 (72.2)	0.627
Time limit (minutes±SD) /No of reporting centers	23 (69.7)	22.73±13.85 / 11/15 (73.3)	20.83±6.33 / 12/18 (66.7)	0.673

**Table 2.** Temperature and Thermal Management in the Delivery Room

	**Total (n=33)**	**Level III maternity hospitals (n=15)**	**Level II maternity Hospitals (n=18)**	**p**
Temperature (T°) of DR °C	33 (100)	24.40±1.05	24.39±1.24	0.978
T° of operating room °C	33 (100)	23.33±2.02	23.83±1.82	0.461
**Thermal management for preterm infants (<32 weeks)**				
Increasing DR T°	13 (39.4)	5/15 (33.3)	8/18 (44.4)	0.386
Preheating the radiant warmer	29 (87.9)	15/15 (100.0)	17/18 (94.4)	0.370
Servo-controlled T°	23 (69.7)	11/15 (73.3)	12/18 (66.7)	0.488
Pre-warmed towels	33 (100)	15/15 (100)	18/18 (100)	NA
Polyethylene plastic bag or wrap	24 (72.7)	11/15 (73.3)	13/18 (72.2)	0.627
Hat	20 (60.6)	10/15 (66.7)	10/18 (55.6)	0.386
Thermal mattress	7 (21.2)	3/15 (20.0)	4/18 (22.2)	0.609
Heated/humidified gases	18 (54.5)	13/15 (86.7)	14/18 (77.8)	0.525
**Thermal management for infants considered at risk of HIE - Passive cooling started:**				
Within 1 hour				
1–2 hours	19 (57.5)	9/15 (60.0)	10/18 (55.6)	-
2–4 hours	2 (6.1)	1/15 (6.7)	1/18 (5.5)	
4–6 hours	12 (36.4)	5/15 (33.3)	7/18 (38.9)	
Mean±SD	33 (100)	2.53±1.96	2.72±1.93	0.783
Range	33 (100)	1–5	1–5	-
Median	33 (100)	1.0	1.5	-

**Table 3.** Umbilical Cord Management

	**Total (n=33)**	**Level III maternity hospitals (n=15)**	**Level II maternity hospitals (n=18)**	**P**
**Umbilical cord management in term infants**				

*Vaginally delivered*				
Physiologically based cord clamping (PBCC)	7 (21.2)	4/15 (26.7)	3/18 (16.7)	0.264
Delayed cord clamping (DCC)	23 (69.7)	8/15 (53.3)	10/18 (55.6)	
Immediate cord clamping (ICC)	2 (6.1)	3/15 (20.0)	4/18 (22.2)	
Milking	1 (3.0)	0/15 (0)	1/18 (5.6)	

*Elective cesarean-delivered*				
PBCC	6 (18.2)	4/15 (26.7)	2/18 (11.1)	0.598
DCC	13 (39.4)	4/15 (26.7)	4/18 (22.2)	
ICC	12 (36.4)	6/15 (40.0)	11/18 (61.1)	
Milking	2 (6.1)	1/15 (6.7)	1/18 (5.6)	

*Emergency cesarean-delivered*				
PBCC	1 (3.0)	0/15 (0)	1/18 (5.6)	0.216
DCC	2 (6.1)	0/15 (0)	2/18 (11.1)	
ICC	26 (78.8)	13/15 (86.7)	13/18 (72.2)	
Milking	4 (12.1)	2/15 (13.3)	2/18 (11.1)	

**Umbilical cord management in preterm infants**				

**Late preterm**				

*Vaginally delivered*				
PBCC	6 (18.2)	4/15 (26.7)	2/18 (11.1)	0.732
DCC	21 (63.6)	5/15 (33.3)	11/18 (55.6)	
ICC	6 (18.2)	6/15 (40.0)	6/18 (33.3)	
Milking	0 (0)	0/15 (0)	0/18 (0)	

*Cesarean-delivered*				
PBCC	4 (12.1)	3/15 (20.0)	1/18 (5.5)	0.970
DCC	11 (33.3)	3/15 (20.0)	5/18 (27.8)	
ICC	15 (45.5)	6/15 (40.0)	12/18 (66.7)	
Milking	3 (9.1)	3/15 (20.0)	0/18 (0)	

**29–32 weeks preterm**				

*Vaginally delivered*				
PBCC	11 (33.3)	6/14 (42.8)	5/18 (27.8)	0.079
DCC	6 (18.2)	5/14 (35.7)	1/18 (5.5)	
ICC	8 (24.2)	1/14 (7.1)	7/18 (38.9)	
Milking	7 (21.2)	2/14 (14.2)	5/18 (27.8)	

*Cesarean-delivered*				
PBCC	7 (21.2)	5/15 (33.3)	2/18 (11.1)	0.051
DCC	8 (24.2)	5/15 (33.3)	3/18 (16.7)	
ICC	12 (36.4)	3/15 (20.0)	9/18 (50.0)	
Milking	6 (18.2)	2/15 (13.3)	4/18 (22.2)	

**<29 weeks preterm**				

*Vaginally delivered*				
PBCC	8 (24.2)	5/15 (33.3)	3/18 (16.7)	0.164
DCC	7 (21.2)	4/15 (26.7)	3/18 (16.7)	
ICC	12 (36.4)	4/15 (26.7)	8/18 (44.4)	
Milking	6 (18.2)	2/15 (13.3)	4/18 (22.2)	

*Cesarean-delivered*				
PBCC	7 (21.2)	5/15 (33.3)	2/18 (11.1)	0.160
DCC	7 (21.2)	4/15 (26.7)	3/18 (16.7)	
ICC	13 (39.4)	3/15 (20.0)	10/18 (55.6)	
Milking	6 (18.2)	3/15 (20.0)	3/18 (16.7)	

**Table 4.** Airway and ventilation in the delivery room

	**Total**	**Level III maternity hospitals (n=15)**	**Level II maternity Hospital (n=18)**	**P**
**Available facilities in hospitals**				
Air/oxygen blender	29 (87.9)	15/15 (100)	14/18 (77.8)	0.075
Pulse oximeter	29 (87.9)	15/15 (100)	14/18 (77.8)	0.075
Heated/humidified gases availability	17 (51.5)	8/15 (53.3)	9/18 (50.0)	0.483
**Initial FiO_2_**				
*≥35-week*				
21%	27 (81.8)	12/15 (80.0)	15/18 (83.3)	-
30%	5 (15.2)	3/15 (20.0)	2/18(11.1)	-
50%	1 (3.0)	0/15 (0)	1 (5.5)	-
Mean±SD	33 (100)	22.80±3.72	23.61±7.19	0.696
Range	33 (100)	21–30	21–100	-
Median	33 (100)	21	21	-
*<35-week infant*				
21%	5 (15.2)	1/15 (6.7)	4/18 (22.2)	-
25–30%	24 (70.7)	14/15 (93.3)	10/18 (55.5)	-
40%	2 (6.1)	0/15 (0)	2/18 (11.1)	-
60%	1 (3.0)	0/15 (0)	1/18 (5.5)	-
100%	1 (3.0)	0/15 (0)	1/18 (5.5)	-
Mean±SD	33 (100)	29.40±2.32	34.31±18.79	0.316
Range	33 (100)	21–30	21–100	-
Median	33 (100)	30	30	-
**PPV**				
Self-inflating bag	12 (36.4)	7/15 (46.7)	5/18 (27.8)	0.514
T-piece device (Neopuff)	21 (63.6)	10/15 (66.7)	11/18 (61.1)	
Mechanical ventilator	0 (0)			
**Ventilatory interface routinely used**				
Facial mask	24 (72.7)	14/15 (93.3)	10/18 (55.5)	0.018
Short binasal prongs	9 (27.3)	1/15 (6.7)	8/18 (44.4)	
Laryngeal mask	4 (12.1)	2/15 (13.3)	2/18 (11.1)	0.626
**The skill of the team on intubation**				
Excellent	16 (48.5)	10/15 (66.7)	6/18 (33.3)	0.141
Good	13 (39.4)	5/15 (33.3)	8/18 (44.4)	
Sufficient	3 (9.11)	0/15 (0)	3/18 (16.7)	
Insufficient	3 (3.0)	0/15 (0)	1/18 (5.5)	
**Sustained lung inflation**				
Yes, routinely	7 (24.2)	2/15 (13.3)	5/18 (27.8)	0.140
Yes, occasionally	8 (21.2)	6/15 (40.0)	2/18 (11.1)	
Never	18 (54.5)	7/15 (46.7)	11/18 (61.1)	
**PIP levels routinely administered to start PPV (cmH_2_O)**				
*Late preterm and term infants*				
<18 cm H_2_O	7 (21.2)	2/15 (13.3)	5/18 (27.8)	0.225
20 cm H_2_O	9 (27.3)	4/15 (26.7)	5/18 (27.8)	
25 cm H_2_O	17 (51.5)	9/15 (60.0)	8/18 (44.4)	
Mean±SD	33 (100)	22.0±3.23	20.72±2.69	
Range	33 (100)	15–25	16–25	
Median	33 (100)	21	20	
*Preterm infants*				
<18 cm H_2_O	14 (42.4)	4/12 (33.3)	10/17 (58.8)	-
20 cm H_2_O	11 (33.3)	3/12 (25.0)	1/17 (5.9)	-
25 cm H_2_O	4 (12.1)	5/12 (41.7)	6/17 (35.3)	-
Mean±SD	33 (100)	20.42±3.39	18.76±3.68	0.230
Range	33 (100)	15–25	8–25	-
Median	33 (100)	20	18	-
**PEEP levels (cmH_2_O)**				
*Late preterm and term infants*				
4	1 (3.0)	0/15 (0)	1/18 (5.5)	-
5	16 (48.5)	9/15 (60.0)	7/18 (38.9)	-
6	14 (42.4)	5/15 (33.3)	9/18 (50.0)	-
7	1 (3.0)	1/15 (6.7)	1/18 (5.5)	-
8	1 (3.0)	0/15 (0)	0/18 (0)	-
Mean±SD	33 (100)	5.47±0.64	5.61±0.85	0.592
Range	33 (100)	5–7	4–8	-
Median	33 (100)	5	6	-
*Preterm infants*				
5	18 (54.5)	8/12 (66.7)	10/17 (58.8)	-
6	10 (30.3)	3/12 (25.0)	7/17 (41.2)	-
7	1 (3.0)	1/12 (8.3)	0/17 (0)	-
8	0 (0)	0/12	0/17	-
Mean±SD	33 (100)	5.42±0.67	5.41±0.50	0.982
Range	33 (100)	5–7	5–6	-
Median	33 (100)	5	5	-
**Meconium-stained amniotic fluid management in a non-vigorous infant**				
Suctioning of the oro- and nasopharynx before delivery of the shoulders	5 (15.2)	2/14 (14.3)	3/18 (16.7)	0.467
Intubation and subglottic aspiration	5 (15.2)	1/14 (7.1)	4/18 (22.2)	
Starting PPV after removing secretions	22 (66.7)	11/14 (78.6)	11/18 (61.1)	

**Table 5.** Circulation, and Medications in the delivery room

	**Total (n=33)**	**Level III maternity (n=15)**	**Level II maternity (n=18)**	**P**
**Heart rate assessment**				
Palpation of the umbilical cord	21 (63.6)	8/15 (53.3)	13/18 (72.2)	**0.224**
Palpation of peripheral pulses	1 (3.0)	0/15 (0)	1/18 (5.5)	**0.545**
Stethoscope	33 (100)	15/15 (100)	18/18 (100)	**NA**
Three-lead ECG monitor	2 (6.1)	0/15 (0)	2/18 (11.1)	**0.290**
Pulse oximeter	22 (66.7)	11/15 (73.3)	11/18 (61.1)	**0.357**
**Surfactant use in delivery room**				
Yes	12 (36.4)	7/15 (46.7)	5/18 (27.8)	**0.224**
LISA	2 (6.1)	1/14 (7.1)	1/18 (5.5)	**0.692**
INSURE	9 (27.3)	6/15 (40.0)	3/18 (16.7)	**0.135**
Caffeine use in delivery room				
Yes	4 (12.1)	2/15 (13.3)	2/18 (11.1)	**0.626**
**Sodium bicarbonate use in delivery room**				
Yes	3 (9.1)	0/15 (0)	3/18 (16.7)	**0.150**
Asphyxia	1 (3.0)	0/15 (0)	1/18 (5.5)	**0.253**
Severe metabolic acidosis	2 (6.1)	0/15 (0)	2/18 (11.1)	

**Table 6.** Education

	**Total (n=33)**	**Level III maternity hospitals (n=15)**	**Level II maternity Hospitals (n=18)**	**P**
Courses on neonatal resuscitation routinely				
6–12 months	20 (60.1)	11/15 (73.3)	9/18 (50.0)	0.157
12–24 months	13 (39.4)	4/15 (26.7)	9/18 (50.0)	
>24 months	0 (0)	0/15 (0)	0/18 (0)	
Neonatal Resuscitation algorithm				
American Academy of Pediatrics (AAP)	28 (84.8)	11/15 (73.3)	17/18 n (94.4)	0.185
European Resuscitation Council (ERC)	3 (9.1)	2/15 (13.3)	1/18 (5.5)	
Romanian Resuscitation Guidelines	3 (9.1)	2/15 (13.3)	1/18 (5.5)	

There were significant differences between level-III and level-II maternity hospitals regarding the number of births in 2018 (3028.73±1258.38 vs 1983.78±769.99; P=0.006), lowest GA of routinely assisted infants in delivery room (25.07±3.03 weeks vs 30.44±3.28, P<0.001), inborn infants with BW<1500 admitted to neonatal intensive care unit (NICU) in 2018 (66.86±39.14 g vs 22.87±31.50 g; P=0.002), and antenatal counseling of parents before the delivery of a very preterm infant or an infant with expected problems (60% vs 22.2%; P=0.027).

The availability of nasal continuous positive airway pressure (CPAP)/high flow nasal canula (HFNC), mechanical ventilation, CPAP in delivery room, the presence of resuscitation team at every delivery, the routinely presence of pediatrician/neonatologist for a low-risk/high risk delivery, and time limit to stop full resuscitation in severely asphyxiated infant were similar between centers. Level-III maternity hospitals were significantly more able to provide high frequency oscillatory ventilation (HFOV) (80% vs. 44.4%; P=0.041), inhaled nitric oxide (iNO) (46.7% vs. 0%; P=0.002), and therapeutic hypothermia (53.3% vs. 11.1%; P=0.012) as compared to level-II maternity hospitals. (Table 1)

Thermal management for preterm infants (<32 weeks) was similar among centers as regards temperature of delivery/operating room and ensuring thermal comfort to avoid heat loss. Regarding thermal management for infants considered at risk of hypoxic-ischemic encephalopathy (HIE), passive cooling was started between 1–2 hours of life both in level-III and level-II maternity hospitals. (Table 2).

There were no significant differences of umbilical cord management regardless of GA between responding centers. (Tabel 3)

No significant differences were identified between responding level II and III centers regarding available equipment designed for effective ventilation during resuscitation. Air/oxygen blenders, pulse oximeters were available in 87.9% of centers, T-piece devices (Neopuff) in 63.6% and heated/humidified gases in the delivery room in only 51.5% of maternity hospitals (level-III 53.3% vs level-II 50%). For positive pressure delivery, both level III and level II centers preferred T-piece device (Neopuff) followed by self-inflating bag. The PIP levels (cmH_2_O) used in term, late preterm, and preterm infants were higher in level-III centers compared with level-II centers, whereas PEEP levels (cmH_2_O) used in term and late preterm infants were higher in level-II centers compared with level-III centers, but the difference was not statistically significant. A PEEP level of 5 cmH_2_O was preferred for preterm infants both in level-III and level-II centers. Initial FiO_2_ was 21% in infants with GA higher than 35 weeks and 30% in preterm infants in all centers. For meconium-stained amniotic fluid management in a non-vigorous infant, starting PPV after removing secretions was the most frequent procedure (66.7%), followed by intubation and subglottic aspiration (15.2%), and suctioning of the oro and nasopharynx before delivery of the shoulders (15.2%), without significant differences between centers. Among technical aspects, fewer level-II resuscitation teams self-evaluated as having excellent skill on performing endotracheal intubation than level-III (Table 4).

All neonatologists are using stethoscopes for heart rate assessment during neonatal resuscitation, 63.6% of them are also using palpation of the umbilical cord, but only 6.1% are using a three-lead ECG monitor, without differences between centers. Surfactant and caffeine administration in delivery room was similar among centers. Sodium bicarbonate was still used in delivery room only in level-II maternities although in a small proportion (16.7%) (Table 5).

Courses and training on neonatal resuscitation are routinely held in all Romanian maternity hospitals, in 60.1% of units at 6–12 months, and in 39.45% at 12–24 months. Most of these courses follow neonatal resuscitation algorithm of American Academy of Pediatrics (AAP) (84.8%) (Table 6).

## Discussion

The Romanian survey evaluated the neonatal resuscitation practices in maternity hospitals with more than 1000 of births per year. Overall, all regions of the country were represented in the study and the results are reflecting an important part of the neonatal level II and III centers in Romania (42.66% of the number of births in 2018). The results demonstrated no major variations in the delivery room management of term and preterm infants between the centers, highlighting a good compliance with international/national guidelines.

### Before birth and ethics

Antenatal counseling of parents before the delivery of a very preterm infant or an infant with expected problems or complications was routinely performed in 39.4% of maternity hospitals, significantly more often in level-III centers who are competent and equipped for caring for the most critical patients. This rate is still lower than the overall rate of European (77%) [5], Italian (90%) [7] and Turkish hospitals (56%) [6]. Antenatal counselling done together by the neonatologist and obstetrician is recommended internationally [9,10]. The counseling should provide objective and realistic information about the risks of morbidity and mortality in extremely preterm infants. The decision on early intensive care (resuscitation and neonatal intensive care) versus palliative comfort care (providing warmth and comfort without medical assistance) should be a shared decision between parents and professionals after counseling. Involving parents in decision making is recommended for the infant’s best interest [11].

The median gestational age for initiating full resuscitation at birth was 24 weeks in our study, the legal age of viability in Romania. The limit of viability has been set at 22–23 weeks (Japan, Germany, Sweden), 23–24 weeks (United Kingdom, USA, Canada), 24–26 weeks (France, Netherlands, Switzerland) [12].

In the Romanian study, the time limit to stop full resuscitation in severely asphyxiated infant was 22.73±13.85 minutes in level-III and 20.83±6.33 minutes in level-II maternity hospitals. According to international guidelines, the individualized decision to continue or discontinue resuscitation should be considered at about 20 minutes after birth if the heart rate remains undetectable and all steps of resuscitation have been completed [13,14].

The presence of a resuscitation team at all high-risk deliveries was similar in Romanian centers. A neonatologist or pediatrician qualified with full resuscitation skills attended 87.9% of low-risk deliveries and 100% of high-risk deliveries, in accordance with resuscitation international guidelines [13,14].

### Temperature and Thermal Management in the Delivery Room

Thermal instability after delivery is a risk factor for increasing morbidity and mortality in infants. Therefore, the maintenance of the infant’s temperature between 36.5°C and 37.5°C is crucial during resuscitation and stabilization at birth. This implies adequate maintenance of ambient temperature at minimum 24°C, use of preheated resuscitation table and towels, servo-controlled temperature, hats, polyethylene plastic bag for preterm infants and heated/humidified medical gases [13,14,15]. According to Romanian survey, the neonatologists are very concerned about preventing heat loss of the in the delivery room. Use of pre-warmed towels (100%), preheating the radiant warmer/resuscitation table (87.9%), polyethylene plastic bag (72.7%) were the most frequent measures instituted among maternity hospital regardless of level of competence. Instead, heated/humidified gases were used in only 54.5% of centers. As the effects of hypothermia are known for infants considered at risk for hypoxic-ischemic encephalopathy (HIE) [16], passive cooling was started in all maternities within the first 6 hours after birth, with more than 50% of units instituting this preventive measure within the first two hours of life.

### Umbilical Cord Management

The timing of cord clamping continues to vary in practice according to local clinical policy. Based on available scientific evidence delayed cord clamping (DCC) - after at least 30–60 seconds after birth - has been shown beneficial in preventing anemia and iron deficiency in full-term infants and in reducing need for blood trans-fusion and decreasing complications, including intraventricular hemorrhage and necrotizing enterocolitis in preterm infants, thus improving outcomes [13,17]. Physiological based cord clamping (PBCC) - sectioning the umbilical cord after lung aeration or initiating respiratory support - is at least as effective as stabilization through DCC [18,19,20]. Immediate cord clamping (ICC) should be considered for cases of maternal hemorrhage, placental abruption, placenta previa. Cord milking may be an alternative to DCC but should be avoided in very preterm infants because complications like brain injury [13].

Our findings show that DCC was provided to more than 60% of vaginally delivered healthy term and late preterm infants in both level-III and level-II centers, similar to reports in other studies [5,6,7]. In term and late preterm infants delivered by elective caesarean section, DCC decreased to less than 40% and in emergency cesarean to less than 7%. In vaginally delivered preterm infants with gestational age between 29–32 weeks, PBCC was preferred, while in caesarean births it prevailed ICC. In newborns with a gestational age of less than 29 weeks, ICC was preferred both for those born vaginally and by caesarean section.

### Airway and ventilation in the delivery room

Effective ventilation is the most important intervention for successful neonatal transition and resuscitation. Hence, the priority is to initiate positive pressure ventilation in the “golden minute” after assessment of need for resuscitation, initial steps, assessment of heart rate/breathing, if the infant is apneic or has a heart rate below 100/min [21]. The Romanian study showed that in both level-III and level-II maternity hospitals there is a high confidence in routinely use of T-piece device (Neopuff) and face mask to administer positive pressure ventilation. It seems that this is a general trend described in other studies as well (5,6,7). Although laryngeal mask is recommended by the International Guidelines [4,14,22] as part of the resuscitation equipment, less than 13% of responders use it when intubation fails. An air-oxygen blender and a pulse oximeter for oxygen titration guiding are available in almost all interviewed Romanian maternity hospitals.

### Circulation, and Medications in the delivery room

A rise in heart rate is the most important indicator of favorable response to resuscitative interventions and effective ventilation is the most important step to correct heart rate below 100/min [21,22]. In both level-III and level-II maternity hospitals the stethoscope is the first choice for evaluating heart rate during resuscitation, while the use of ECG monitor - as the International Guidelines are recommending - had low adherence, probably due to lack of equipment. Sodium bicarbonate is still used in delivery room, only level-II centers, apparently only in documented situations.

### Education

Neonatal resuscitation teams must continuously train and adapt to the latest evidence-based resuscitation guidelines. The skills and behaviors needed to perform effectively are acquired through continuous and simulation-based training. All maternity hospitals reported that retraining is done more frequently than every 2 years, which is optimal. Romanian Neonatology Association developed a national guideline for neonatal resuscitation in accordance with international guidelines and play an important role in organizing courses, workshops, summer-schools on this topic. The neonatal resuscitation program (NRP) is a part of training healthcare professionals carrying for newborns.

### Strengths and limitations of the study

The representativity of the sample was satisfactory, given that responding centers reported on 42.66% of all Romanian births in 2018. The overall response rate to the questionnaire was 62.26%, higher in level-III (83.33%) and limited in level-II (51.43%). Maternity hospitals that report less than a thousand births per year were not considered because the European questionnaire was designed only for hospitals with more than a thousand births per year. For a more accurate picture of the resuscitation practices in Romania, we should also include in the study the level-I maternities hospitals that do not have neonatal intensive care (NICU) in their structure.

## Conclusions

The results of the Romanian survey are reflecting the implementation stage of international and national guidelines of neonatal resuscitation in maternity hospitals with more than 1000 births per year. The adherence to new guidelines was high among responding centers regarding thermal and umbilical cord management, initial FiO_2_, but aspects like antenatal counseling, EKG monitoring, laryngeal mask, and heated/humidified gases availability and administration, and simulation-based training require further implementation.
